# Reliability and Construct Validity of the Work Rehabilitation Questionnaire Domains in Patients with Persistent Low Back Pain

**DOI:** 10.1007/s10926-024-10248-1

**Published:** 2024-11-08

**Authors:** Anders Hansen, Henrik Hein Lauridsen, Reuben Escorpizo, Karen Søgaard, Jens Søndergaard, Berit Schiøttz-Christensen, Ole Steen Mortensen

**Affiliations:** 1 Medical Research Unit, Spine Centre of Southern Denmark, Lillebaelt Hospital, University Hospital of Southern Denmark, Østre Houghvej 55, 5500 Middelfart, Denmark; 2 Department of Regional Health Research, University of Southern Denmark, Odense, Denmark; 3 Department of Sports Science and Clinical Biomechanics, University of Southern Denmark, Odense, Denmark; 4 Department of Rehabilitation and Movement Science, The University of Vermont, Burlington, VT USA; 5 Research Unit for General Practice, Department of Public Health, University of Southern Denmark, Odense, Denmark; 6 Department of Occupational and Social Medicine, Holbæk Hospital, Part of Copenhagen University Hospital, Holbæk, Denmark; 7 Department of Public Health, Section of Social Medicine, University of Copenhagen, Copenhagen, Denmark

**Keywords:** Clinimetric, Work functioning, Physical functioning, Psychological well-being, Cognitive ability

## Abstract

**Purpose:**

The Work Rehabilitation Questionnaire (WORQ) assesses patient functioning, including psychological, physical, and cognitive limitations. This study evaluates the WORQ domains in individuals with persistent low back pain (LBP), focusing on reliability and construct validity.

**Methods:**

Individuals aged 18–65 with LBP completed WORQ and the workability index single item. A subgroup undertook sit-to-stand and 6-min walking tests and re-evaluated WORQ after 14 days. Reliability was assessed through internal consistency (McDonald’s omega and Cronbach’s alpha), test–retest reliability, and smallest detectable change. Construct validity was analyzed via Spearman’s rank correlation and known group validity, with physical functioning also examined against sit-to-stand and 6-min walk test results for sensitivity/specificity. Floor and ceiling effects were assessed through classical and scale width methods.

**Results:**

Of 425 participants, 149 completed physical tests, and 102 re-assessed WORQ. McDonald’s omega and Cronbach’s alpha indicated high internal consistency (0.92–0.96) with strong test–retest reliability (intraclass-correlation coefficients: 0.74–0.82). The smallest detectable change ranged from 4.62 to 7.82. Predictions from 7 out of 8 hypotheses were confirmed. Notable differences in domain scores were observed based on disability level and sick leave status, with varied diagnostic performance in physical functioning items. Potential floor effects were noted using the scale width method.

**Conclusions:**

The WORQ demonstrated good reliability and satisfactory validity in assessing work-related functioning in individuals with persistent LBP. These findings support its use as a comprehensive tool for evaluating psychological, physical, and cognitive limitations. However, varied diagnostic performance in physical functioning items and potential floor effects suggest cautious interpretation in diverse clinical settings.

## Introduction

Low back pain (LBP) is a frequent condition that negatively impacts workforce productivity and places a substantial financial strain on healthcare systems. The importance of addressing LBP is emphasized by projections indicating that it will impact approximately 834 million individuals worldwide by 2050 [1], underscoring its role as a significant factor in work-related absenteeism.

LBP affects various facets of life, including physical, mental, and social functions. Understanding its impact on work functioning is complex and necessitates a comprehensive framework considering health and functioning as well as context-related factors. The International Classification of Functioning, Disability, and Health (ICF) framework [2] by the World Health Organization can be used for this purpose. It is a biopsychosocial model that considers functioning as a product of the interaction between the individual’s health condition and contextual factors, such as personal traits and the environment. Existing outcome assessment tools for LBP, such as the Oswestry Disability Index (ODI) [3] and the Roland-Morris Disability Questionnaire [4], primarily focus on physical functioning. However, they often lack a comprehensive evaluation of psychological well-being and cognitive ability, which are important for understanding the multifaceted impact of LBP on work functioning. The Work Rehabilitation Questionnaire (WORQ) was developed in alignment with the ICF framework [5] and offers a holistic perspective on individuals’ impairments and disabilities. It is intended for various patient populations and facilitates the identification of specific areas requiring targeted and person-centered interventions. The validity of the WORQ has been assessed in diverse populations, including patients with musculoskeletal disorders, spinal cord injury, and those entering vocational rehabilitation, demonstrating its versatility and robustness [6–10]. Additionally, the WORQ has demonstrated excellent test–retest reliability, with studies reporting high ICC values, underscoring its reliability in measuring work-related functioning over time [7, 9]. In a recent assessment of its structural validity in patients with LBP, three key domains emerged: “physical functioning,” “psychological well-being,” and “cognitive ability” [11]. This study evaluates the statistical robustness of these domains and their clinical relevance. The emphasis on clinical applicability reflects our commitment to refining the WORQ beyond statistical metrics to enhance person-centered interventions. Thus, the present study aims to extend the assessment of WORQ in patients with LBP by investigating the reliability and construct validity of the domains.

## Methods

The trial was conducted at the Spine Center of Southern Denmark, an outpatient medical department dedicated to spine diagnostic. Patients are referred to this center by general medical practitioners, chiropractors, or hospital departments if the initial therapeutic interventions fail to yield improvement.

Following the Consensus-based Standards for Selecting Health Measurement Instruments (COSMIN) guideline [12], reliability and construct validity were evaluated. Reliability is the degree to which the measurements are free from measurement error, while construct validity is the degree to which the scores of a measurement instrument are consistent with hypotheses, e.g., regarding internal relationships, relationships with scores of other instruments, or differences between relevant groups.

## Study Design

The study was conducted from December 2021 to December 2022 and focused on persons between 18 and 65 years with persistent LBP. We identified eligible participants using self-reported data from “My Spine Data” (MiRD), which builds upon the SpineData registry [13]. We excluded individuals not active in the labor market and those who rated their likelihood of working within the next six months as 0 (on a scale of 0–10, where 0 = no likelihood and 10 = total likelihood).

Following informed consent, participants completed the self-administered WORQ and the single-item Work Ability Index (WAI) electronically. They also performed the 6-min walk test (6MWT) and a 30-s sit-to-stand (STS) test to evaluate their functional capacity. Fourteen days after inclusion, they were asked to electronically reassess their WORQ scores, ensuring they had not undergone any significant diagnostic procedures or invasive treatments, like MRI scans or surgery. The re-evaluation phase and functional capacity assessments were discontinued after 100 repeat WORQ scores had been collected. The decision was based on achieving a statistically robust sample size for test–retest reliability and correlation assessments [14] while optimizing resource utilization.

### Patient-Reported Information

Participants’ demographic (age, sex, body mass index) and clinical characteristics, including LBP intensity, leg pain intensity, disability, health status, and if insurance claims were filed, were assessed using self-reported data from the MiRD database. Pain intensity was assessed using a numeric rating scale (0–10), where participants reported their average pain over the past week [15]. Patient functioning and health state included information from the Oswestry Disability Index (ODI) and the EuroQol-5D (EQ-5D-5L) [16]. The ODI comprises ten items assessing disability. Each item presents six response categories in a Likert-like format (0 = no disability, 5 = severe disability). The sum score is transformed linearly to 0–100, with high scores indicating a higher disability [3]. The EQ-5D-5L is an instrument that assesses health status. It comprises five dimensions (Mobility, Self-Care, Usual Activities, Pain/Discomfort, and Anxiety/Depression) with five response levels and a visual analog scale (0–100) rating overall health [16]. These self-reports were provided within a week before inclusion. The ODI and EQ-5D-5L were chosen based on their established reliability and validity. The ODI is widely used to measure disability related to LBP and evaluate its impact on daily activities [3]. The EQ-5D-5L assesses general health status across five dimensions, capturing the broad impact of health conditions [16]. These tools were favored for their ease of administration and extensive use in clinical and research settings, ensuring comparability with other studies.

The WORQ encompasses 40 items distributed across three domains: psychological well-being (items: 4, 5, 6, 7, 8), physical functioning (items: 12, 13, 14, 15, 22, 27, 28, 30, 31, 32), and cognitive ability (items: 9, 10, 17, 18, 19, 20, 23, 24, 25, 26). Additionally, it includes 15 screening questions (items: 1, 2, 3, 11, 16, 21, 29, 33, 34, 35, 36, 37, 38, 39, 40). Each item is scored on an 11-point numeric rating scale (0–10 where 0 = no problems or difficulty to 10 = significant problems / extreme difficulty). To facilitate interpretation, both domain and single-item scores are linearly transformed into a 0 to 100 scale, with higher scores indicating more significant functional limitations and disabilities [5]. The questionnaire can be acquired at www.myworq.org.

The WAI single item is a self-reported measure of the participant’s current work ability compared to their lifetime best, rated on a 0 to 10 numeric rating scale (where 0 = completely unable to work to 10 = work ability at its best”) [17].

## Physical Capacity Tests

The 6MWT took place in a quiet 30-m hallway, with participants covering as much ground as possible in 6 min [18]. A stopwatch was employed to time the test, and the assessor recorded the number of turnarounds (every 30 m). After 6 min, the distance covered on the course was measured with a laser range finder (DeWalt DW033) and recorded to the nearest 0.1 m. The result was computed by summing the partial course length distance and the number of turnarounds.

For the 30-s STS, a standard chair with a height of 43 cm was used. Participants aimed to rise to a standing position as many times as possible within 30 s, with arms crossed on their chests. Individuals unable to perform a transfer in a pretrial assessment were excluded from the test. The score reflects the number of transfers completed within 30 s, considering a transfer as complete if it was more than halfway done by the 30th second [19].

The 6MWT and STS were chosen based on their ability to quantitatively evaluate walking capacity and lower limb strength, which serve as indicators of physical functioning [18, 19].

### Statistical Analyses

Demographic and clinical information was summarized using descriptive statistics. Demographic differences were assessed between participants who underwent functional capacity tests and those who did not.

### Reliability Measures

The internal consistency of WORQ scales was assessed using McDonald’s omega coefficient and Chonbach’s alpha. Omega and alpha values ≥ 0.7 indicate that a significant portion of the variance is attributed to the underlying construct rather than natural fluctuations, indicating reliable and homogeneous measurement [20]. Test–retest reliability was determined by the intraclass correlation coefficient (ICC) by the two-way mixed-effect ANOVA model with interaction for absolute agreement. Results were categorized into moderate (0.5–0.74), good (0.75–0.9), and excellent (> 0.9) reliability levels [14].

Analysis of baseline and retest data aimed to evaluate measurement errors, including both systematic and random errors. The standard error of measurement (SEMagreement) was calculated as SDpooled × √(1 − ICCagreement) [21]. The smallest detectable change (SDCagreement), denoting the minimum change required to assert a difference beyond natural fluctuation confidently, was determined as 1.96 × √2 × SEMagreement and reported with 95% CIs [21].

### Validity Measures

The construct validity of the WORQ domains was evaluated by correlating them with well-established work functioning and health status measures, including the WAI, the EQ-5D-5L, and physical capacity tests such as the 6MWT and the STS test. We hypothesized a moderate negative correlation between the psychological well-being domain and the WAI single item (expected < − 0.3), drawing on a demonstrated inverse relationship between psychological distress and work capacity, with higher distress predicting more absences from work and lower job satisfaction [22]. Furthermore, a strong positive correlation was expected between psychological well-being and the EQ-5D-5L item for anxiety/depression (expected > 0.5), as both constructs are associated with mental health and well-being, with previous findings indicating that psychological distress can significantly affect an individual’s perceived health status [23].

In relation to the physical functioning domain, we predicted strong negative correlations with the STS, 6MWT, and WAI single item (< − 0.5). These correlations are grounded in the understanding that the ability to complete STS and walking movements is a composite measure of lower extremity muscle performance, including factors such as strength, balance, and speed, which are essential for physical work capacity [24].

For the cognitive ability domain, we hypothesized a moderate negative correlation with the WAI single item (expected < − 0.3) and positive correlations with EQ-5D-5L item 5 (Anxiety/Depression) and the ODI item 7 for sleep function (both expected > 0.3). This is supported by literature indicating that cognitive impairments and sleep disturbances have an impact on work functioning [25, 26].

These hypothesized correlations (low: 0.1–0.3, moderate: 0.31–0.5, and strong: >  = 0.51) [27] were tested using Spearman’s rank correlation coefficient. Consistency with these predictions would affirm the WORQ’s construct validity, with the benchmark set by Terwee et al.’s [28] criteria requiring confirmation of at least 75% of the hypotheses.

### Sensitivity, Specificity, and Predictive Values

The diagnostic performances of the physical functioning domain and the 6MWT and STS were assessed. The WORQ domain scores were dichotomized into “low” or “high” by sum scores of ‘0–49’ and ‘50–100,’ respectively. This dichotomy facilitated cross-tabulation calculations involving sensitivity, specificity, and positive and negative predictive values. The scores for the 6MWT and STS were divided into “low” and “high” by the lower and upper tertiles. The choice to divide functional test scores into tertiles rather than halves was made to capture more nuanced distinctions in performance. We only used the “low” and “high” tertiles of the functional capacity test to ensure true “low” and “high” functional capacity measures. Additionally, individual items within the physical functioning domain were analyzed and categorized as “low” (scores 0–4) or “high” (scores 5–10).

### Known Group Validity

Group validity was evaluated using the Wilcoxon Rank-Sum test on domain scores. This included comparing participants on and off sick leave or reporting high and low disability (defined by an ODI cutoff score of >  = 40% (17)).

### Floor/Ceiling Effects

Floor and ceiling effects were evaluated using two methods: the classical method and the scale width method. The classical method involves calculating the percentage of participants who scored at the lowest or highest possible end of the scale. Specifically, a floor effect is observed if a significant portion of participants scores at the minimum value, while a ceiling effect is observed if many participants score at the maximum value. The scale width method assesses the ability of the scale to detect meaningful changes over time by examining the distribution of scores within the SDC range at both extremes of the scale [29]. The floor and ceiling effects are considered acceptable if no more than 15% of participants have scores within the SDC range at the lower or upper limits of the scale. All statistical analyses were made in R version 4.3.3 [30] using the “psych” package [31].

## Results

### Participant Recruitment

A total of 532 patients consented to the study, with 425 (80% response rate) completing the WORQ and WAI. One hundred and forty-nine (35%) performed the STS and 6MWT, and 102 (24%) re-scored the WORQ after 14 days.

**Fig. 1:**
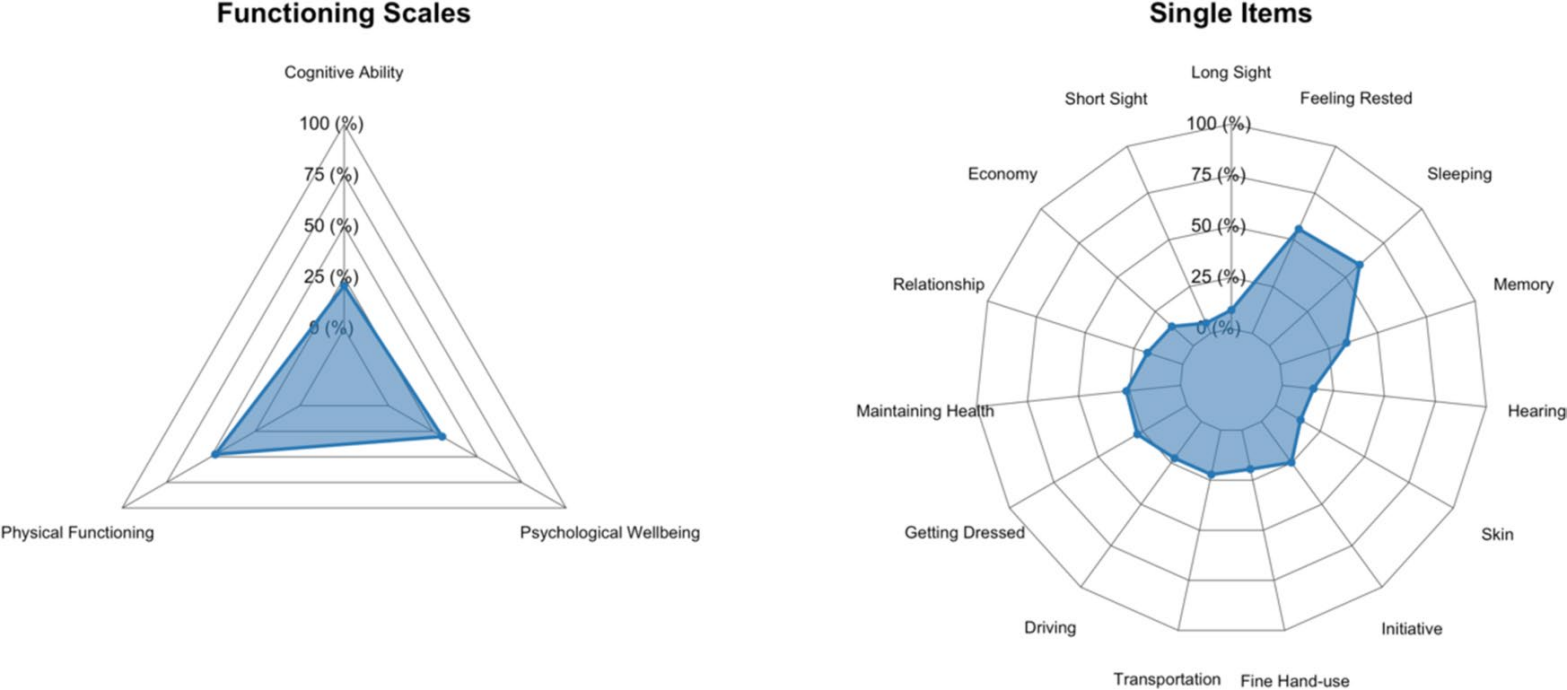
Radar plot—domains and single item

Notably, despite a negligible difference in gender composition, there were no statistically significant variations in demographic or self-reported data between participants who completed the WORQ and those who did not (see Table 1). Additionally, no differences were observed between patients who performed capacity tests and those who did not (data not shown). Figure 1 visually presents the sample’s baseline distribution of mean scores across the domains and functioning items. Each axis represents a different domain, with the plotted values indicating the average level of impairments or functional limitations reported by participants. Higher scores reflect greater difficulty within that scale or item.

**Table 1:** Demographic characteristics and differences between participants and non-participants

Characteristic	Participants, *N* = 425^a^	Non-participants, *N* = 107^a^	*p* value^b^
Age	53 (43, 59)	50 (39, 58)	0.2
Sex, female	269 (63%)	53 (52%)	0.05
BMI^c^	27.6 (24.3, 31.7)	27.4 (24.1, 31.6)	0.8
LBP intensity past week	5 (4, 7)	5 (3, 7)	0.4
Leg pain intensity past week	4 (1, 6)	3 (1, 5)	0.2
ODI^d^	30 (20, 40)	28 (18, 38)	0.3
Insurance claim filed	44 (10%)	10 (9.9%)	0.9
EQ5D vas^e^	55 (39, 74)	59 (40, 70)	> 0.9

^a^Median (Interquartile range); *n* (%)

^b^Wilcoxon rank sum test; Pearson’s chi-squared test

^c^Oswestry Disability Index

^d^Body mass index

^e^EQ-5D-5L (EuroQol-5D)

### Internal Consistency and Reliability Assessment

The WORQ domains had robust internal consistency, with omega and Cronbach’s alpha values of 0.95 and 0.92 for psychological well-being, 0.94 and 0.92 for physical functioning, and 0.96 and 0.94 for cognitive ability. As indicated by the ICC, reliability was good. Specifically, ICCs were 0.75 (95% CI 0.65; 0.82) for psychological well-being, 0.82 (95% CI 0.75; 0.88) for physical functioning, and 0.74 (95% CI 0.64; 0.82) for cognitive ability.

### Measurement Error Assessment

For the psychological well-being domain, which ranges from 0 to 50, we observed an SEM of 0.85 and an SDC of 4.62 (95% CI 2.95; 6.29). In the physical functioning domain ranging from 0 to 100, the SEM was 1.39, and the SDC was 7.55 (95% CI 4.83; 10.3). For the cognitive ability domain ranging from 0 to 100, the SEM was 1.44, with an SDC of 7.82 (95% CI 5.0; 10.6).

### Hypothesis Testing

The study achieved an 87.5% accuracy in predicting hypotheses, with 7 out of 8 confirmed (Table 2).

**Table 2:** Hypothesis testing in construct validity of WORQ scales

Items or tests	WORQ scales	Correlation^a^		Correctly predicted	
		Expected	Observed	Yes	No
WAI_single item_	Psychological well-being	< − 0.3	− 0.41	√	
EQ5D_item5_ (anxiety/depression)	Psychological well-being	> 0.5	0.53	√	
WAI_single item_	Physical functioning	< − 0.5	− 0.66	√	
STS^b^	Physical functioning	< − 0.5	− 0.64	√	
6MWT^c^	Physical functioning	< − 0.5	− 0.52	√	
WAI_single item_	Cognitive ability	< − 0.3	− 0.41	√	
EQ5D_item5_ (anxiety/depression)	Cognitive ability	> 0.3	0.45	√	
ODI_item7_ (sleep function)	Cognitive ability	> 0.3	0.26		√

^a^Spearman’s Rho

^b^Sit-to-stand test

^c^6-min walk test

*WAI*  Work Ability Index, *EQ5D* EuroQol-5D, *ODI* Oswestry Disability Index

### Indicators of Accuracy for WORQ

Table 3 outlines the diagnostic performance measures for the physical functioning domain and its specific items related to STS and 6MWT. Notably, the domain shows limitations in accurately identifying true positives and true negatives, as indicated by the low sensitivity and specificity values. The low positive predictive values and negative predictive values also indicate challenges for the domain to accurately predict the capacities of positive and negative test results.

**Table 3:** Calculation of sensitivity, specificity, positive, and negative values based on dichotomised WORQ physical functioning scale items, Sit-to-stand tests, and the 6-min walk test

WORQ domain/item	6MTW^a^				STS^b^			
	Sensitivity	Specificity	PPV^c^	NPV^d^	Sensitivity	Specificity	PPV^c^	NPV^d^
Physical functioning	0.36	0.27	0.33	0.29	0.32	0.14	0.28	0.17
12	0.40	0.12	0.32	0.17	0.44	0.21	0.37	0.26
13	0.06	0.61	0.14	0.39	0.06	0.73	0.19	0.43
14	0.08	0.53	0.15	0.36	0.08	0.61	0.17	0.39
15	0.31	0.37	0.33	0.35	0.30	0.39	0.33	0.35
22	0.50	0.13	0.37	0.19	0.52	0.27	0.42	0.35
27	0.44	0.20	0.36	0.26	0.44	0.27	0.29	0.32
28	0.28	0.33	0.30	0.31	0.24	0.41	0.29	0.34
30	0.60	0.04	0.37	0.10	0.66	0.10	0.41	0.24
31	0.16	0.18	0.17	0.18	0.24	0.29	0.26	0.27
32	0.10	0.42	0.15	0.31	0.16	0.55	0.27	0.39

^a^6-min walk test

^b^Sit-to-stand test

^c^Positive predicted value

^d^Negative predicted value

In the analysis of individual items within both assessments, sensitivity displayed variability. Its lowest value was observed for item 13 (bodily pain), while its highest value was noted for item 30 (short-distance walking). Conversely, specificity demonstrated an inverse pattern. Positive predictive values varied across items, with the least associations seen for items 13 (bodily pain), 14 (endurance), 31 (long-distance walking), and 32 (mobility), and the highest for item 30 (short-distance walking). Similarly, negative predictive values also showed variability across items, with the lowest association found for item 22 (day-to-day activities) and 30 (short-distance walking) and the highest for item 13 (bodily pain).

### Known Group Validity

**Fig. 2:**
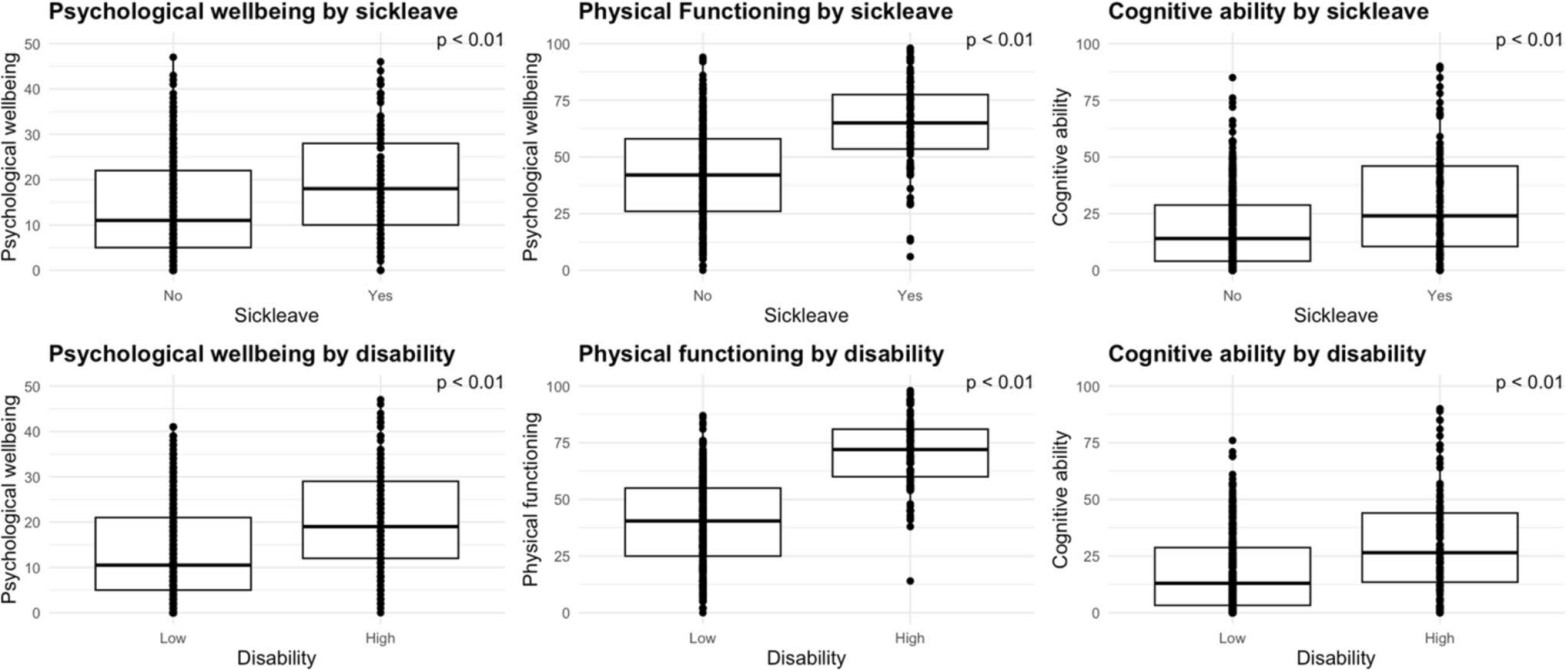
Known-group validity between WORQ domains and patients on and off sick leave and high or low disability. WORQ scores range from 0 to 100, with higher scores indicating greater severity of functional limitations. Disability cutoff score (ODI ≥ 40)

The WORQ domains demonstrated robust known group validity by effectively differentiating participants based on disability levels and sick leave status. In the psychological well-being domain, those with higher self-rated disability or on sick leave scored 12.6% and 17.4% higher, respectively, compared to individuals with low self-rated disability or working. In the physical functioning domain, participants experiencing high disability and sick leave scored 32.1% and 22.5% higher, respectively. Within the cognitive ability domain, individuals with increased disability and on sick leave scored 14.5% and 12.6% higher than their counterparts, see Fig. 2.

### Floor and Ceiling Effect

**Fig. 3:**
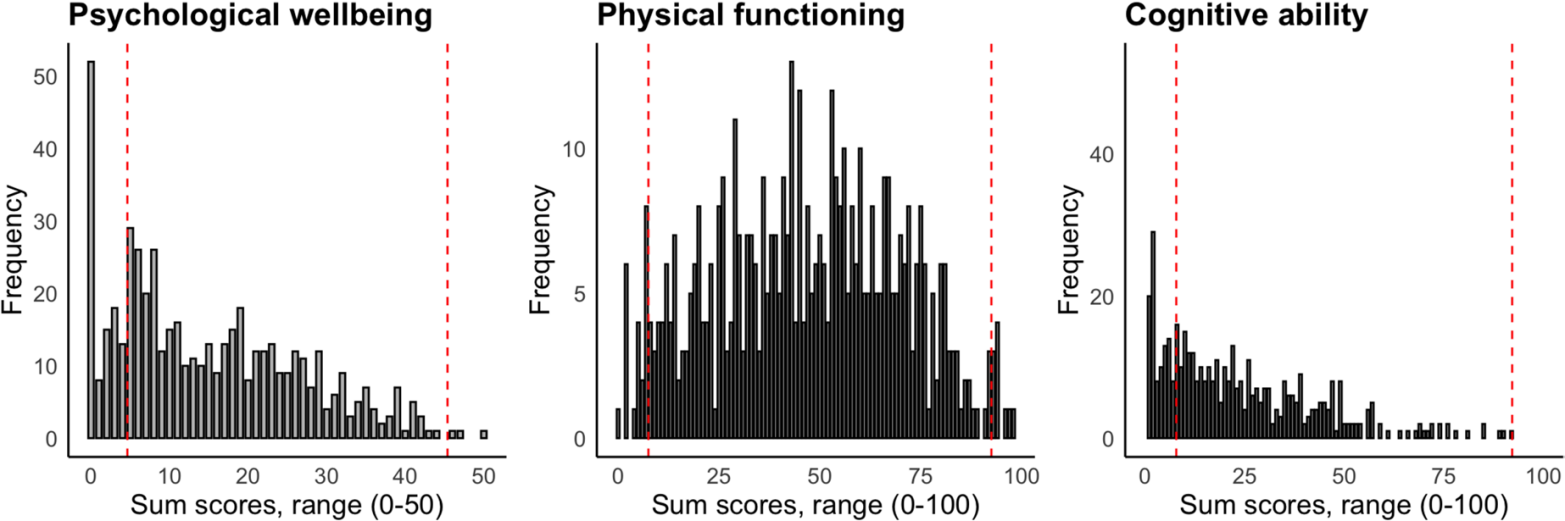
Raw WORQ domain scores. Dashed lines = SDC_95%_

The evaluation of floor and ceiling effects revealed notable limitations in the scales’ sensitivity to mild impairments in specific domains. For the psychological well-being domain, the classical method indicated a floor effect of 9.9% and a negligible ceiling effect of 0.2%, suggesting challenges in detecting mild psychological impairments. This was further emphasized by the scale width method, which showed a substantial floor effect of 20%, indicating potential inadequacies in capturing milder psychological issues outside of measurement error. Similarly, the cognitive ability domain had comparable floor effects; the classical method reported a floor effect of 9.9%, while the scale width method highlighted a more pronounced effect of 30.1%. These results raise concerns about the domain’s ability to detect mild cognitive difficulties, suggesting that further investigation may be warranted to assess and potentially refine the sensitivity of the measurement scales, especially within the psychological well-being and cognitive ability domains. In contrast, the physical functioning domain displayed minimal floor and ceiling effects across both methods, indicating a more effective range of functioning scores. Refer to Table 4 and Fig. 3 for the domain-specific floor and ceiling effects, including statistics.

**Table 4:** Floor and ceiling

	Classical method		Scale width		
	Floor (%)	Ceiling (%)	Floor (%)	Ceiling (%)	Range
Psychological well-being	9.9	0.2	20	0.5	0–50
Physical functioning	0.3	0.3	3.1	3.1	0–100
Cognitive ability	9.9	0.3	30.1	0.0	0–100

## Discussion

This study measured the reliability and construct validity of the WORQ domains in a cohort of patients with persistent LBP. High internal consistency and good test–retest reliability were observed across the domains, alongside minimal measurement error. Hypothesis testing supported the WORQ’s ability to evaluate work functioning and discriminate between patient subgroups based on disability and sick leave status. Key findings highlighted the sensitivity and specificity of the WORQ physical functioning domain and its items. While some items displayed moderate sensitivity and specificity, others demonstrated limited discriminative capacity. The positive and negative predictive values indicated varying degrees of precision in predicting impairment. The distribution analysis revealed a tendency toward lower scores in the psychological well-being and cognitive ability domains, indicating a significant floor effect. Such effects necessitate cautious interpretation of scores, especially in cases of mild impairment. Although these findings suggest a limitation in the WORQ’s range for detecting less severe issues, the instrument’s overall utility in a clinical context remains supported by its strong reliability and validity metrics, as previously reported [5–10, 32, 33].

### Reliability

The WORQ domains’ high internal consistency supports its reliability and is consistent with the psychometric properties expected of a robust clinical assessment tool. Notably, the domains’ internal consistency suggests that the items within each domain appropriately capture a unidimensional construct of LBP. Hence, the WORQ domains can reliably capture functioning in patients with persistent LBP.

The smallest detectable change values observed across the domains indicate the extent of measurement error, providing a benchmark for identifying true change at the 95% confidence level among the LBP population. The SDC for cognitive ability in our LBP population was lower than reported in a study involving patients with various musculoskeletal conditions [8]. This suggests that the measurement error for cognitive ability in our study is less pronounced, potentially reflecting the unique characteristics and impairments of the LBP population. For physical functioning and psychological well-being domains, comparisons are not directly feasible because the reported SDC values pertain to the unique factor structure of the LBP population. This underscores the need for domain-specific evaluations within contextually relevant populations.

While the test–retest reliability observed is clinically relevant for interpreting group-level outcomes in a clinical context over time, they do not meet the recommended threshold of an ICC greater than 0.9 for individual-level decisions [34], suggesting caution for individual patient management. Although this study did not evaluate the Minimal Important Change (MIC), the established reliability and construct validity provide a foundation for future inquiries into the MIC’s determination for individual patient care.

### Construct Validity

The analysis demonstrated statistically significant correlations between WORQ domains and the WAI single item, particularly in the physical functioning domain, which showed the strongest correlation. This supports the hypothesis that enhancing physical functioning could reduce work disability in individuals with LBP [35]. However, it is also critical to acknowledge the substantial, though less pronounced, influence of psychological well-being and cognitive ability on workability. Additionally, the substantial correlation between psychological well-being and anxiety/depression highlights the WORQ’s capacity to discern key elements of psychological health, implying that improving psychological well-being may have beneficial effects on overall health outcomes. Furthermore, strong correlations between the physical functioning scale and the STS test and the 6MWT reinforce the validity of this domain in assessing physical function.

While the correlation between cognitive ability and sleep function was low, it was anticipated due to the interrelation [36]. Furthermore, it is important to interpret this finding with caution. Cognitive ability likely has a more direct association with work-related functioning, and using ‘sleep function’ as a proxy may not comprehensively capture cognitive aspects. Practical constraints influenced this decision in available cognitive assessment tools for standard LBP evaluations. It underscores the imperative for future research to explore the intricate relationship between cognitive ability, pain conditions, workability, and the need for more refined assessment.

The correlation between cognitive ability and anxiety/depression (item 5 of EQ-5D-5L) was medium, indicating the effectiveness of the cognitive ability domain in capturing psychological aspects of work functioning. It is important to note that while the EQ-5D-5L item on anxiety/depression is a validated tool and widely used, it is a single-item measure and may not capture the full complexity of anxiety and depression as comprehensively as multi-item scales. Nonetheless, the findings align with previous research, identifying a robust correlation between the cognitive ability domain and depression and anxiety [8]. Furthermore, the strong correlation between the psychological well-being domain of the WORQ and anxiety/depression highlights the sensitivity of the psychological well-being domain in detecting psychological distress. The consistent associations across both cognitive ability and psychological well-being domains with anxiety and depression measures reinforce the WORQ’s utility in providing a comprehensive assessment of work-related functioning, capturing both cognitive and psychological dimensions.

The diagnostic performance evaluation of the physical functioning domain score and the ten domain items revealed significant variability. Some items had high sensitivity and specificity, while others were less effective. This variation implies that certain items are better suited for specific diagnostic purposes, while others may not be as accurate in identifying physical impairments in individuals with LBP. Notably, item 13 (bodily pain) demonstrated high specificity in both the STS and the 6MWT assessments, suggesting that the absence of pain reliably indicates the absence of impairments in lower leg strength or limitations in walking, making it a valuable tool for ruling out such conditions. Conversely, item 30 (short-distance walking) had high sensitivity in the assessments, indicating that self-rated walking restriction effectively identifies individuals with actual walking limitations. These findings suggest that the WORQ may not be uniformly effective across all aspects of measuring physical functioning, but it excels as a screening tool. However, it is important to consider that the questionnaire’s positive and negative predictive values may vary depending on the target population’s characteristics and prevalence and cannot be extrapolated beyond patients with persistent LBP.

Our analysis found relatively low positive and negative predicted values across all items within the physical functioning scale. Positive predicted values assess how accurately a high severity score corresponds to high physical functioning levels, while negative predicted values assess the accuracy of a low severity score in indicating low physical functioning levels. This suggests that an individual’s physical capabilities are influenced by various factors beyond what is captured by physical measurements alone.

In the ICF framework, specific qualifiers are used to rate the extent of functional decline. Body and structure impairment determine presence and severity, while functional performance and capacity qualifiers assess activity difficulties and participation restrictions [2]. The distinction between performance and capacity is important in this context. Performance reflects a person’s actions influenced by environmental factors in their current surroundings, typically assessed through patient-reported outcomes or task performance in their actual environment. On the other hand, capacity refers to an individual’s ability to be evaluated in a controlled environment, accounting for environmental adjustments, as seen in assessments like the 6MWT and STS. The relationship between self-reported performance and actual capacity levels in individuals with LBP has been studied, with existing literature reporting diversities [37, 38]. This may partly explain the low predictive values for using self-reports to predict capabilities, underscoring the complexity of evaluating functional abilities. The disparity between what individuals believe they can do and what they achieve in various situations highlights the need for a comprehensive assessment of physical functioning, ideally incorporating both subjective patient-reported assessments and standardized capacity measurements.

The predominance of lower scores in the psychological well-being and cognitive ability scales of the WORQ suggests that, while frequently encountered in persistent LBP, the severity of issues varies. Clinicians should consider these dimensions for their potential impact on functionality and quality of life, recognizing that low scores could indicate widespread mild issues or significant problems in a subset of patients. Nevertheless, the observed floor effects for these domains at 20% for psychological well-being and 30.1% for cognitive ability may reflect a lack of sensitivity in detecting less severe impairments. These effects could potentially obscure the domains’ responsiveness to change, a consideration vital for both clinical practice and future research. Addressing the scale’s sensitivity to change, especially when dealing with subtle yet clinically important shifts in patient status, is important in developing comprehensive assessment tools in LBP management. However, it is important to note that when the WORQ is used as a screening tool, the presence of floor effects is less critical. The primary goal of a screening tool is to identify individuals who may have significant impairments and require further evaluation or intervention. Therefore, the ability to detect mild impairments, while useful, is not essential for the WORQ's intended purpose as a screening instrument. Alternatively, persons with mild impairment or limitation may not require rehabilitation services but may rather be provided patient education and preventive strategies.

### Strengths and Limitations

The methodological strength of this study is enhanced by using a rule-of-thumb principle of 10 responses per questionnaire item. Although this approach is in line with conventional practices, it is important to recognize that sample size estimation involves multiple considerations, and the adequacy of the sample size remains uncertain. The large sample, nonetheless, improves the representativeness of the results and reduces non-response bias through detailed demographic and self-reported analyses of non-respondents versus respondents. The non-responder analysis strengthens the assertion that selection bias is likely minimal. Another strength is the use of patient-reported outcomes and capacity-based measures, both commonly used in clinical settings. However, a significant limitation is exclusive reliance on self-report for establishing eligibility, which, while practical, may not capture all dimensions of the condition. Another key limitation is using suboptimal proxies, like sleep function, to assess cognitive ability, revealing a need for more refined cognitive assessment tools in future studies to better evaluate this complex relationship.

## Conclusion

The WORQ showed good reliability and validity for assessing functional limitations in a cohort of individuals with persistent LBP, as evidenced by consistently reliable outcomes across various measures and strong construct validity. The observed risk of floor effects in the psychological and cognitive domains raises concerns, particularly when evaluating changes over time. The results support the practical utility of the WORQ in clinical settings, but caution is advised for longitudinal assessments as responsiveness and MIC have yet to be established.

## Data Availability

The datasets generated during and/or analyzed during the current study are available from the corresponding author upon reasonable request.
